# Investigation of pyrophosphate versus ATP substrate selection in the *Entamoeba histolytica* acetate kinase

**DOI:** 10.1038/s41598-017-06156-5

**Published:** 2017-07-19

**Authors:** Thanh Dang, Cheryl Ingram-Smith

**Affiliations:** Department of Genetics and Biochemistry and the Eukaryotic Pathogens Innovation Center, Clemson University, Clemson, SC 29634 USA

## Abstract

Acetate kinase (ACK; E.C. 2.7.2.1), which catalyzes the interconversion of acetate and acetyl phosphate, is nearly ubiquitous in bacteria but is present only in one genus of archaea and certain eukaryotic microbes. All ACKs utilize ATP/ADP as the phosphoryl donor/acceptor in the respective directions of the reaction (acetate + ATP ${\boldsymbol{\leftrightarrows }}$ acetyl phosphate + ADP), with the exception of the *Entamoeba histolytica* ACK (EhACK) which uses pyrophosphate (PP_i_)/inorganic phosphate (P_i_) (acetyl phosphate + P_i_ ${\boldsymbol{\leftrightarrows }}$ acetate + PP_i_). Structural analysis and modeling of EhACK indicated steric hindrance by active site residues constricts entry to the adenosine pocket as compared to ATP-utilizing *Methanosarcina thermophila* ACK (MtACK). Reciprocal alterations were made to enlarge the adenosine pocket of EhACK and reduce that of MtACK. The EhACK variants showed a step-wise increase in ADP and ATP binding but were still unable to use these as substrates, and enzymatic activity with P_i_/PP_i_ was negatively impacted. Consistent with this, ATP utilization by MtACK variants was negatively affected but the alterations were not sufficient to convert this enzyme to P_i_/PP_i_ utilization. Our results suggest that controlling access to the adenosine pocket can contribute to substrate specificity but is not the sole determinant.

## Introduction

Acetate kinase (ACK; E.C. 2.7.2.1) catalyzes the reversible transfer of phosphate from acetyl phosphate to a phosphoryl acceptor (S), yielding acetate and a phosphorylated product (S-P) [Eq. 1]. ACK, nearly ubiquitous in bacteria, has been identified in just a single genus of archaea, *Methanosarcina*. In recent years, ACK has also been identified in certain eukaryotic microbes including the green algae *Chlamydomonas*, euascomycete and basidiomycete fungi, and certain protists, namely *Entamoeba histolytica* ^1^.

1 $${\rm{Acetyl}}\,{\rm{phosphate}}+{\rm{S}}\leftrightarrows {\rm{acetate}}+{\rm{S}} \mbox{-} {\rm{P}}$$

This enzyme was discovered in 1944^2^ and the first kinetic characterization was reported in 1954^3^. In 2001, Buss *et al*. solved the structure for the *Methanosarcina thermophila* ACK (MtACK), and subsequent studies with this archaeal enzyme determined that ACK proceeds through a direct in-line mechanism of phosphoryl transfer^4–6^. Acyl substrate selection in ACK has been studied in the *Methanosarcina* enzyme. Four key residues, Val^93^, Leu^122^, Phe^179^, and Pro^232^, have been shown to form a hydrophobic pocket for acetate binding^7^ implicated in acyl substrate selection in this enzyme. In particular, Val^93^ appears to play an important role in limiting substrate length.

Ordinarily, ACK utilizes ATP/ADP as phosphoryl donor/acceptor; however, the *E. histolytica* enzyme is unusual in that it is PP_i_-dependent. Instead of using ATP/ADP as the phosphoryl donor/acceptor [Eq. 2], *E. histolytica* ACK (EhACK) can only use pyrophosphate (PP_i_)/inorganic phosphate (P_i_) as the phosphoryl donor/acceptor [Eq. 3]^8–10^.

2 $${\rm{Acetyl}}\,{\rm{phosphate}}+{\rm{ADP}}\leftrightarrows {\rm{acetate}}+{\rm{ATP}}$$

3 $${\rm{Acetyl}}\,{\rm{phosphate}}+{{\rm{P}}}_{{\rm{i}}}\leftrightarrows {\rm{acetate}}+{{\rm{PP}}}_{{\rm{i}}}$$

whereas most ATP-dependent ACKs function in both directions of the reaction, the *E. histolytica* enzyme strongly prefers the acetate-forming direction^8–10^. Double reciprocal plots of substrate concentration versus enzyme activity indicated EhACK follows a ternary-complex mechanism^8–10^, supporting a direct in-line mechanism of phosphoryl transfer as seen for MtACK^4–6^. Currently, EhACK is the only known ACK to utilize pyrophosphate or inorganic phosphate as a phosphoryl donor or acceptor instead of ATP.

ACK belongs to the ASKHA (acetate, sugar kinase, heat shock and actin) enzyme superfamily. In 1992, Bork *et al*. identified PHOSPHATE1, PHOSPHATE2 and ADENOSINE as three signature ATPase motifs shared by members of this superfamily^11^. These three conserved motifs form part of the adenosine binding pocket and are directly involved in ATP binding. Thaker *et al*. solved the EhACK structure and noted two amino acids substitutions in the ADENOSINE motif versus MtACK that may sterically hinder ATP binding^12^.

Here, we investigated the role of residues in the ADENOSINE and PHOSPHATE2 motifs in phosphoryl substrate selection and utilization in ACK. Our results indicated that the adenosine pocket and the ADENOSINE motif play a critical role in ATP binding. However, ATP binding alone did not lead to utilization. Thus, although EhACK shares strong similarities with ATP-dependent ACKs, subtle differences have dramatically shaped its identity and function.

## Results

Structures have been solved for six bacterial (four of which are from *Mycobacterium*), one archaeal, and two eukaryotic ACKs^4, 12–14^. Although the global structures are similar, the percent identity and similarity between these ACK sequences showed that the eukaryotic ACKs are less related to the bacterial and archaeal enzymes (Supplemental Table S1). Previous phylogenetic analysis revealed that fungal ACKs belong to a distinct clade but the *E. histolytica* and other eukaryotic sequences group with the bacterial and archaeal ACKs^1^. Thus, the unique P_i_/PP_i_-dependence of EhACK must be due to localized differences in the active site.

### Structural differences between the adenosine binding pocket of PP_i_-and ATP-ACKs

**Figure 1:**
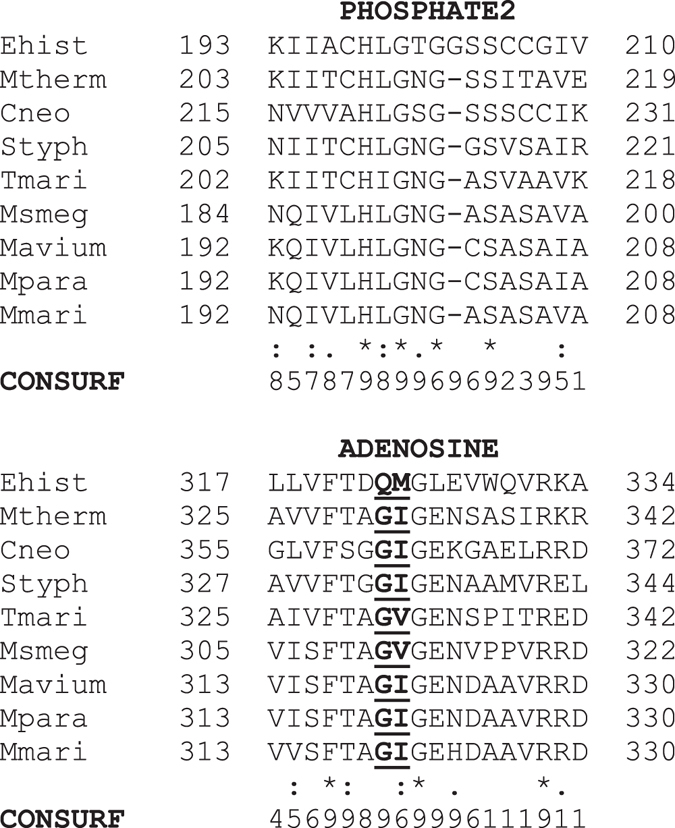
Partial alignment of ACK amino acid sequences. Sequences of ACKs for which the structure have been solved were aligned and ConSurf analysis was performed to examine sequence conservation. The PHOSPHATE2 and ADENOSINE motifs are shown. The full alignment is provided in the Supplemental Information (Supplemental Figure S2). Abbreviations and PDB accession numbers: Ehist, *E. histolytica*, PDB ID 4H0O; *Methanosarcina thermophila*, PDB ID 1TUY; Cneo, *Cryptococcus neoformans*, PDB ID 4H0P; Styph, *Salmonella typhimurium*, PDB ID 3SLC; Tmari, *Thermotoga maritima*, PDB ID 2IIR; Msmeg, *Mycobacterium smegmatis*, PDB ID 4IJN; Mavium, *Mycobacterium avium*, PDB ID 3P4I; Mpara, *Mycobacterium paratuberculosis*, PDB ID 3R9P; Mmari, *Mycobacterium marinum* PDB ID 4DQ8.

In addition to the PHOSPHATE1, PHOSPHATE2, and ADENOSINE sequence motifs, Ingram-Smith *et al*.^15^ defined two other regions designated as LOOP3 and LOOP4 that also influence ATP binding in ACK. Inspection of the active site in the MtACK structure showed that these regions surround the ATP binding site, with ADENOSINE forming a hydrophobic pocket for the adenosine moiety of ADP/ATP^4, 7, 10, 12, 15^. ConSurf analysis (http://consurf.tau.ac.il), which estimates the evolutionary conservation at each position based on phylogenetic and structural analysis, indicated that the central positions in ADENOSINE have the highest conservation level (Fig. 1). Positions 322–327 of EhACK are of particular note as this region of the ADENOSINE motif is strongly conserved in other ACKs but not EhACK.

**Figure 2:**
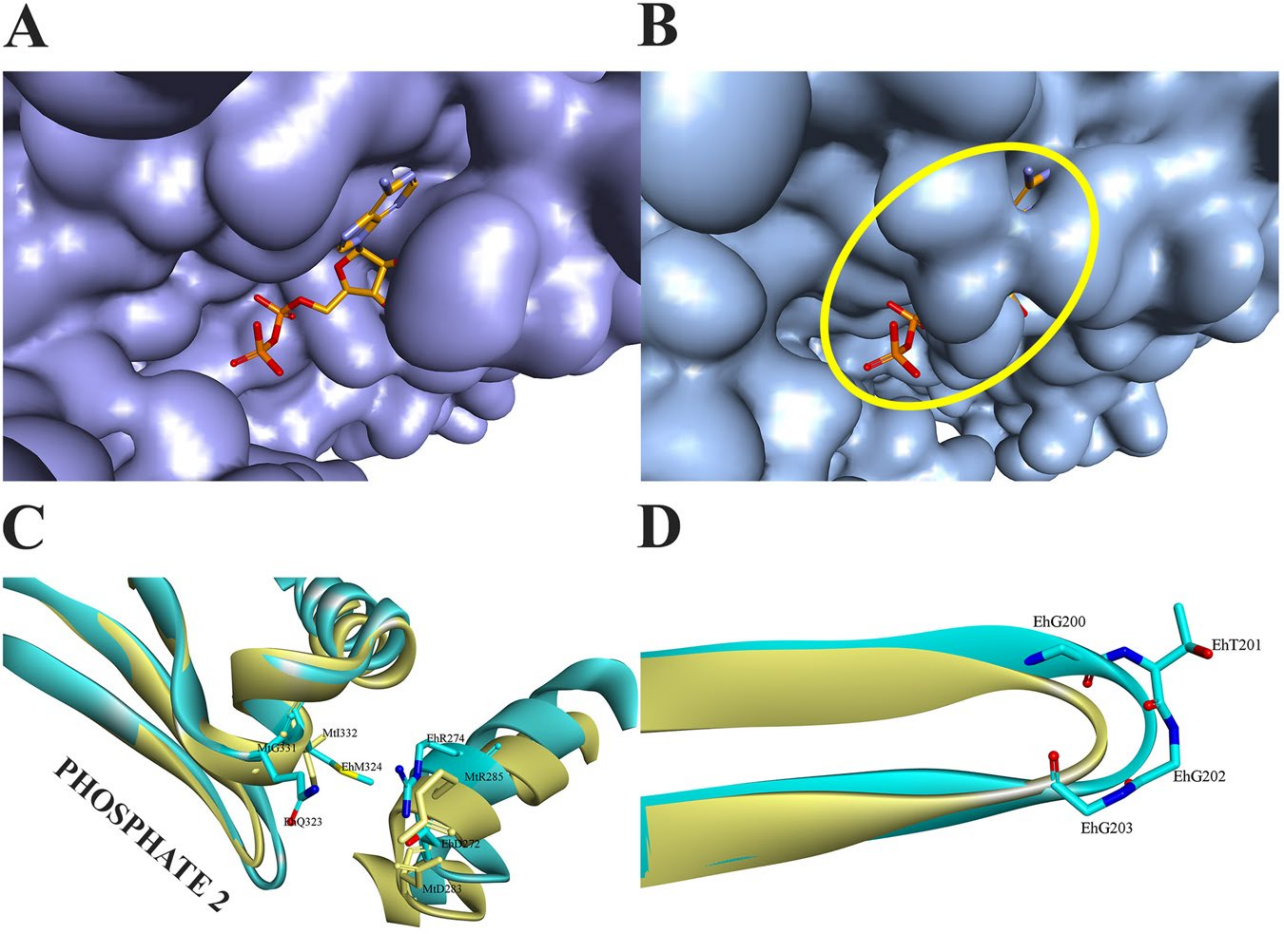
The ACK adenosine binding pocket. (**A**) Surface representation of MtACK with bound ADP. (**B**) Surface representation of EhACK. The constricted opening to the adenosine pocket is circled. The position of bound ADP in MtACK was superimposed into the EhACK structure. (**C**) Superimposition of the adenosine pocket from MtACK (yellow) and EhACK (cyan) showing the positions of targeted residues. (**D**) Superimposed PHOSPHATE2 motifs from MtACK (yellow) and EhACK (cyan), showing the position of the additional Gly in the EhACK PHOSPHATE2 motif.

ADP/ATP-utilizing ACKs have a highly conserved Gly residue and an adjacent Ile/Val within the ADENOSINE motif (positions 331 and 332 in MtACK). Inspection of the MtACK structure revealed a large opening to the adenosine binding pocket (Fig. 2A) that is also evident in the structures of other ATP-ACKs. Occlusion of the adenosine pocket is evident in the surface representation of EhACK (Fig. 2B). Thaker *et al*.^12^ postulated that Gln^323^Met^324^ within the ADENOSINE motif of EhACK may sterically prevent ADP/ATP binding.

### Role of the ADENOSINE motif in ATP/ADP versus PP_i_/P_i_ utilization

To investigate the role of the ADENOSINE motif in determining substrate selection, EhACK variants were created that simulate the open adenosine pocket observed in ATP-dependent ACKs. Gln^323^ and Met^324^ were altered to Gly and Ile, respectively, to mimic the residues found at equivalent positions in MtACK. These positions were also both altered to Ala to minimize side chain intrusions into the opening of the adenosine pocket. The reverse alterations were made in MtACK, converting Gly^331^-Ile^332^ to Gln-Met, respectively, to determine the effect of closing the entry to the adenosine pocket. The EhACK and MtACK variants were purified (Supplemental Figure S1) and kinetic parameters were determined in both directions of the reaction (Table 1).

**Table 1:** Apparent kinetic parameters for wild-type and variant EhACKs and MtACKs.

Enzyme	Substrate	*K*_*m*_ (mM)	*k*_cat_ (sec^−1^)	*k*_cat_ */K*_*m*_ (sec^−1^ mM^−1^)
**EhACK**		**Acetate-forming direction**		
Wild-type	AcP	0.57 ± 0.03	266 ± 12	467 ± 44
Q^323^G-M^324^I		0.47 ± 0.04	32 ± 1.0	68 ± 7.5
Q^323^A-M^324^A		0.34 ± 0.07	14 ± 1.2	42 ± 5.3
G^203^deletion-Q^323^G-M^324^I		No detectable activity		
D^272^A-R^274^A-Q^323^G-M^324^I		0.66 ± 0.11	0.052 ± 0.005	0.08 ± 0.01
Wild-type	P_i_	14 ± 1.2	196 ± 5	14 ± 1.7
Q^323^G-M^324^I		7.3 ± 0.5	28 ± 1.2	3.8 ± 0.35
Q^323^A-M^324^A		8.2 ± 1.1	20 ± 0.8	2.5 ± 0.22
G^203^deletion-Q^323^G-M^324^I		No detectable activity		
D^272^A-R^274^A-Q^323^G-M^324^I		18 ± 1.2	0.076 ± 0.004	0.004 ± 0.0002
		**Acetyl phosphate-forming direction**		
Wild-type	Acetate	166 ± 7	1.4 ± 0.05	0.0086 ± 0.0004
Q^323^G-M^324^I		372 ± 40	0.51 ± 0.02	0.0014 ± 0.0001
Q^323^A-M^324^A		566 ± 44	0.51 ± 0.03	0.0009 ± 0.0001
G^203^deletion-Q^323^G-M^324^I		No detectable activity		
D^272^A-R^274^A-Q^323^G-M^324^I		No detectable activity		
Wild-type	PP_i_	2.1 ± 0.33	0.90 ± 0.03	0.44 ± 0.060
Q^323^G-M^324^I		2.0 ± 0.37	0.45 ± 0.03	0.23 ± 0.030
Q^323^A-M^324^A		2.3 ± 0.41	0.36 ± 0.01	0.16 ± 0.033
G^203^deletion-Q^323^G-M^324^I		No detectable activity		
D^272^A-R^274^A-Q^323^G-M^324^I		No detectable activity		
**MtACK**		**Acetate-forming direction**		
Wild-type	AcP	1.2 ± 0.09	1550 ± 54	1294 ± 56
G^331^Q-I^332^M		1.7 ± 0.14	12 ± 0.7	7.2 ± 0.16
Wild-type	ADP	1.8 ± 0.13	1915 ± 15	1073 ± 69
G^331^Q-I^332^M		5.7 ± 0.56	12 ± 0.5	2.0 ± 0.14
		**Acetyl phosphate-forming direction**		
Wild-type	Acetate	20 ± 0.3	789 ± 16	39 ± 1.0
G^331^Q-I^332^M		302 ± 11	17 ± 4.2	0.057 ± 0.016
Wild-type	ATP	1.7 ± 0.03	711 ± 23	421 ± 10
G^331^Q-I^332^M		9.4 ± 0.27	14 ± 0.2	1.5 ± 0.054

The Q^323^G-M^324^I and Q^323^A-M^324^A EhACK variants displayed similar *K*_*m*_ values for acetyl phosphate and slightly decreased *K*_*m*_ values for phosphate as the unaltered enzyme but the *k*_cat_ values were decreased 8.3-fold for the Q^323^G-M^324^I variant and 19-fold for Q^323^A-M^324^A variant, resulting in ~7 and 11-fold reduced catalytic efficiency, respectively. In the direction of acetyl phosphate formation, these variants displayed slightly increased *K*_*m*_ for acetate but no increase in *K*_*m*_ for PP_i_ and only mild decrease in *k*_cat_. No activity was observed with either variant using ATP or ADP as substrate in the respective direction of the reaction.

The G^331^Q-I^332^M alteration in MtACK resulted in substantial reductions in *k*_cat_ (Table 1). In the acetate-forming direction, catalysis was reduced over 100-fold, and in the acetyl phosphate-forming direction, *k*_cat_ was reduced ~50-fold. This alteration resulted in ~5-fold increase in *K*_*m*_ for ADP and ATP, and a 15-fold increase in *K*_*m*_ for acetate but no substantial change in the *K*_*m*_ for acetyl phosphate. As for wild-type MtACK, no activity was observed with P_i_ or PP_i_ as substrate in the respective directions of the reaction.

### Additional structural elements may contribute to occlusion of the ATP/ADP binding pocket

A salt bridge between Arg^274^ and Asp^272^ on LOOP4 of EhACK may cause further constriction of the adenosine pocket by positioning the Arg side chain in toward the pocket^12^. These two residues are conserved in MtACK but the side chain of Arg is positioned away from the adenosine pocket. These residues are not conserved in among all ACKs though and LOOP4 does not impinge upon the adenosine pocket. The PHOSPHATE2 motif, which interacts with the β phosphate of ATP and was suggested to have a role in substrate positioning^4, 11, 15^, is longer in EhACK and protrudes farther into the active site than in the ATP-dependent enzymes (Fig. 2C). Sequence alignment and structural superposition of the PHOSPHATE 2 motif illustrate that this difference arises from addition of a single residue, Gly^203^ (Figs 1 and 2D).

To examine whether this salt bridge and the extended PHOSPHATE2 motif influence substrate selection, EhACK Q^323^G-M^324^I variants in which the salt bridge has been eliminated (D^272^A-R^274^A-Q^323^G-M^324^I) or in which the PHOSPHATE2 motif has been shortened (ΔG^203^- Q^323^G-M^324^I) were analyzed. The D^272^A-R^274^A-Q^323^G-M^324^I replacement decreased *k*_*cat*_ in the acetate-forming direction by ~2,500–5,000 fold but had little effect on *K*_*m*_ for either substrate (Table 1). This variant had no detectable activity in the acetyl phosphate-forming direction (Table 1). The ΔG^203^- Q^323^G-M^324^I variant was inactive in either direction of the reaction, and thus the effect of the Gly^203^ deletion compounded onto the D^272^A-R^274^A-Q^323^G-M^324^I alteration was not examined. No enzymatic activity was observed with either of these variants using ATP or ADP as the substrate in place of PP_i_ or P_i_.

### Inhibition of EhACK and MtACK by alternative phosphoryl donors and acceptors

**Figure 3:**
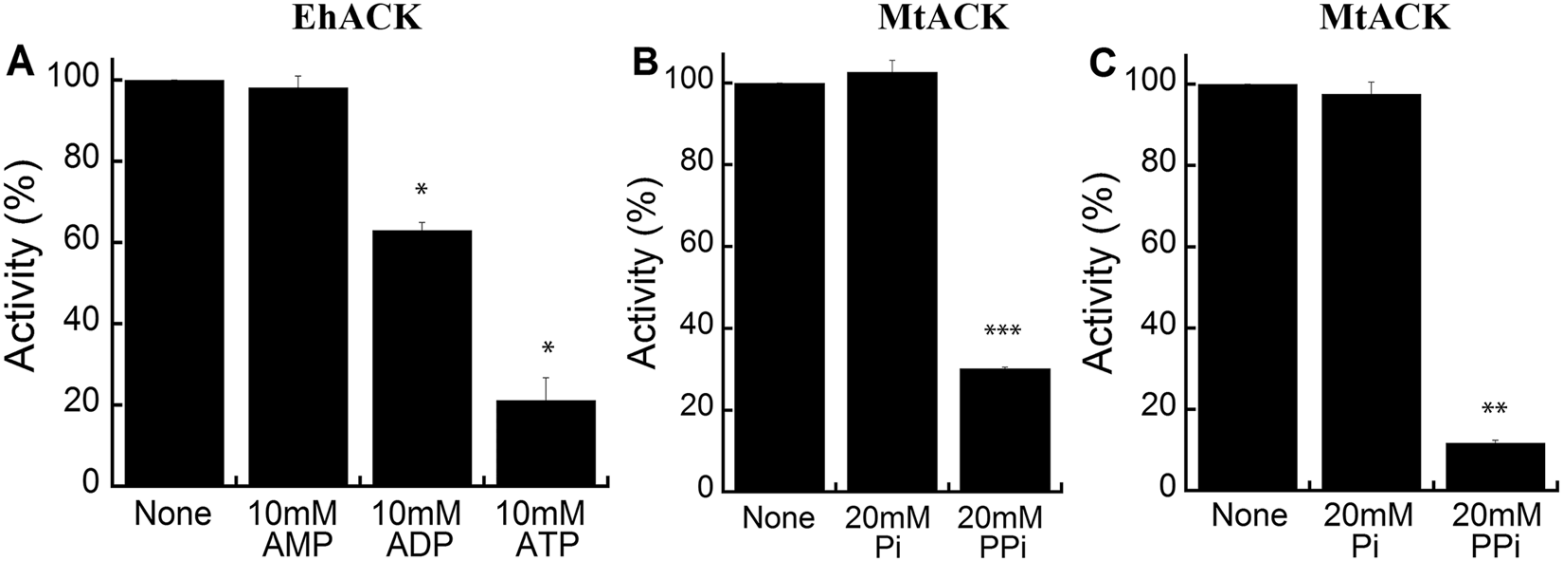
Inhibition of EhACK and MtACK by alternative phosphoryl donors and acceptors. (**A**) Inhibition of wild-type EhACK in the presence of 10 mM AMP, ADP, or ATP in the acetate-forming direction of the reaction. (**B**) Inhibition of wild-type MtACK in the presence of 20 mM P_i_ or PP_i_ in the acetate-forming direction. (**C**) Inhibition of wild-type MtACK in the presence of 20 mM P_i_ or PP_i_ in the acetyl phosphate-forming direction. Significant difference between inhibitions compared to wild-type enzyme activity is tested using an unpaired Welch t-test with R. *p-value < 0.001, **p-value < 0.00003, ***p-value < 0.000008. Activities are the mean ± SD of three replicates.

Since wild-type EhACK and MtACK cannot utilize ATP/ADP or PP_i_/P_i_, respectively, as alternative phosphoryl donor/acceptor, inhibition assays were performed to determine whether these compounds can bind and inhibit activity even if they cannot be used productively as substrate. EhACK activity was measured in the favored acetate-forming direction in the presence or absence of 10 mM AMP, ADP, or ATP (Fig. 3A). Although AMP had no effect, both ADP and ATP were found to inhibit EhACK activity but to differing extents. The presence of ATP resulted in nearly 80% inhibition versus ~30% inhibition by ADP. The presence of P_i_ or PP_i_ with AMP was not sufficient to mimic the effect of inhibition by ADP or ATP, respectively (data not shown). For MtACK, P_i_ had no effect on enzymatic activity. PP_i_ inhibited the enzyme in both directions of the reaction to differing extents, producing ~70% inhibition in the acetate-forming direction and ~90% inhibition in the acetyl phosphate-forming direction (Fig. 3B and C).

**Figure 4:**
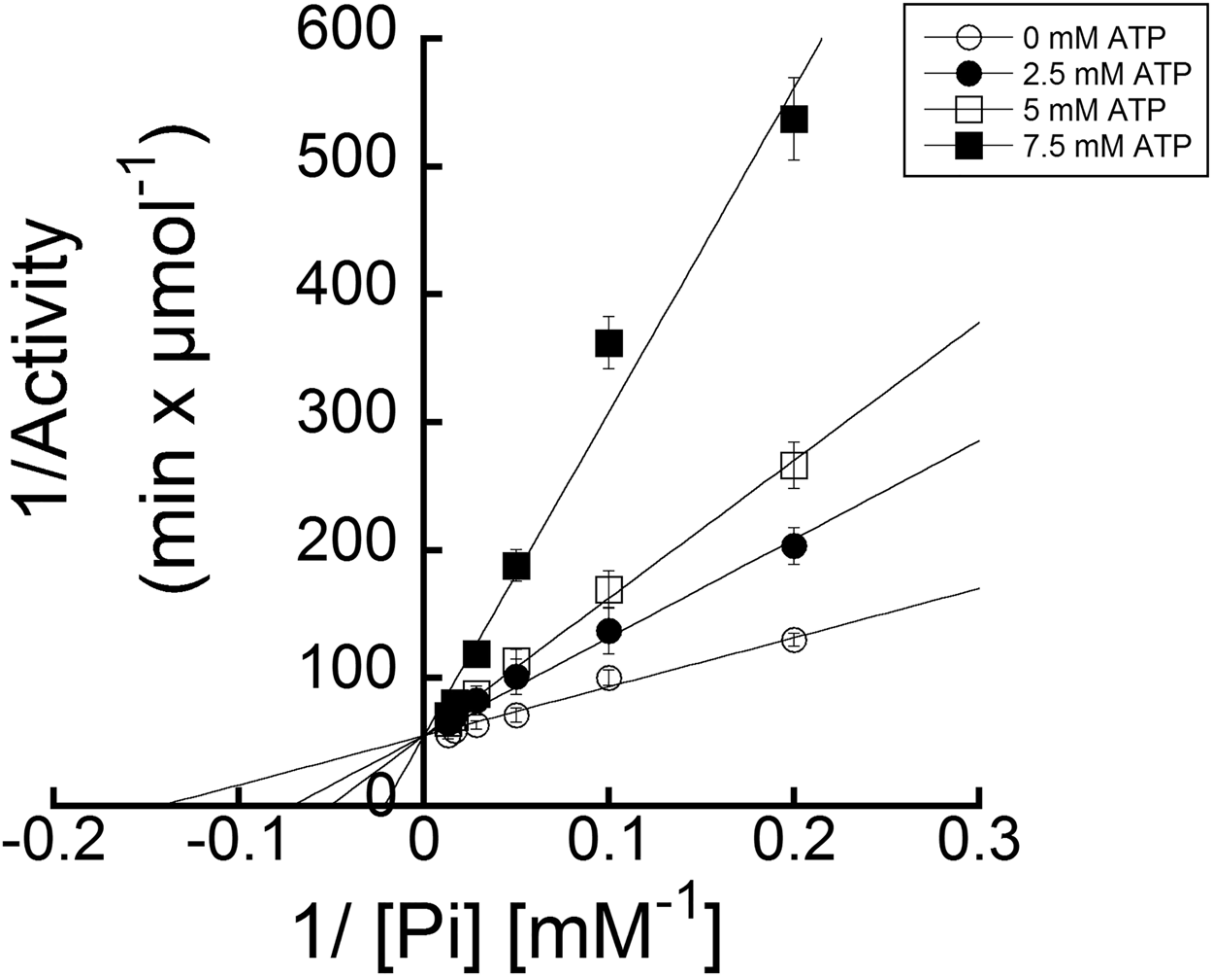
ATP is a competitive inhibitor of EhACK. Double reciprocal plot of EhACK activity versus P_i_ concentration in the absence (○) or presence of 2.5 mM (•), 5 mM (□), or 7.5 mM ATP (■). Activities are the mean ± SD of three replicates.

**Figure 5:**
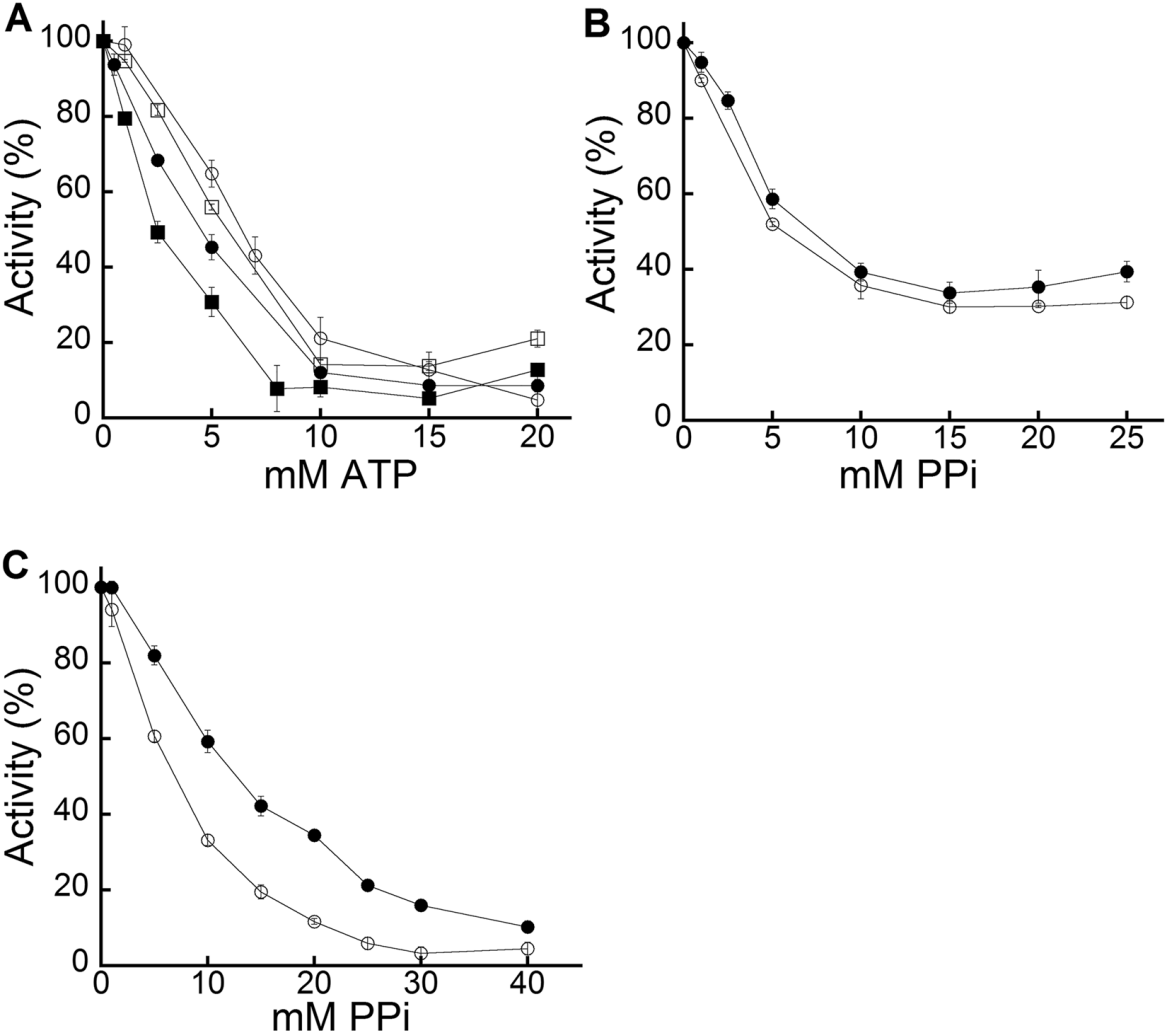
Inhibition curves for EhACK and MtACK wild-type and variant enzymes. Enzymatic activity was determined for each enzyme in the presence of the indicated concentration of ATP or PP_i_. Activities were plotted as a percentage of the activity observed for the wild-type enzyme in the absence of inhibitor. Activities are the mean ± SD of three replicates. (**A**) ATP inhibition of EhACK and its variants in the acetate-forming direction. EhACK wild-type, (○); EhACK Q^323^G-M^324^I variant (•); EhACK Q^323^A-M^324^A variant, (□); EhACK D^272^A-R^274^A-Q^323^G-M^324^I variant (■). (**B** and **C**) PP_i_ inhibition of MtACK and its variants in the acetate-forming (**B**) and acetyl phosphate-forming (**C**) directions. MtACK wild-type, (○); MtACK G^331^Q-I^332^M variant (•).

The mode of inhibition of EhACK by ATP was determined by kinetic analysis using a matrix of reactions in which the ATP concentration was varied versus P_i_ concentration with the acetate concentration held constant. ATP was found to be a competitive inhibitor of EhACK, as demonstrated by the results in Fig. 4. Further examination of ATP inhibition of the EhACK variants in the favored acetate-forming direction of the reaction revealed that a similar final level of inhibition of ~85–90% was achieved for the variants and wild-type enzyme by 15 mM ATP (Fig. 5A).

The IC_50_ value for ATP, defined as the concentration of ATP required to cause 50% inhibition of enzymatic activity, was reduced for the EhACK variants versus the wild-type enzyme (Table 2). The IC_50_ values were reduced 20–30% for the two Q^323^-M^324^ variants versus the wild-type enzyme, whereas the quadruple variant in which both the Q^323^-M^324^ residues and the D^272^-R^274^ salt bridge were altered had an IC_50_ value that was reduced by over 60%. Since ATP is a competitive inhibitor of EhACK activity, this increase in inhibition suggests that the D^272^A-R^274^A-Q^323^G-M^324^I variant binds ATP more efficiently than wild-type enzyme even though it cannot use it as a substrate.

**Table 2:** IC_50_ values for ATP inhibition of EhACK and PP_i_ inhibition of MtACK.

EhACK	ATP IC_50_ (mM)	
	Acetate-forming direction	
Wild-type	6.1 ± 0.05	
Q^323^G-M^324^I	4.2 ± 0.30	
Q^323^A-M^324^A	4.8 ± 0.09	
D^272^A-R^274^A-Q^323^G-M^324^I	2.4 ± 0.19	
**MtACK**	**PP**_**i**_ **IC**_**50**_ **(mM)**	
	**Acetate-forming direction**	**Acetyl phosphate-forming direction**
Wild-type	3.3 ± 0.06	7.5 ± 0.28
G^331^Q-I^332^M	4.0 ± 0.28	14 ± 1.1

ND, not determined because the enzyme was inactive in this direction.

For MtACK, inhibition by PP_i_ in the acetate-forming direction was similar for the wild-type enzyme and the G^331^Q-I^332^M variant, with ~70% maximum inhibition observed (Fig. 5B). The IC_50_ values for PP_i_ were similar for both enzymes (Table 2). PP_i_ inhibition in the acetyl phosphate-forming direction was much stronger, reaching greater than 90% for both the wild-type and variant enzymes. However, maximum inhibition for the wild-type enzyme was achieved at lower ATP concentration (20–25 mM versus 40 mM for the variant). This is reflected in the IC_50_ value, which is nearly two-fold higher for the G^331^Q-I^332^M variant than for the wild-type.

## Discussion

### Substrate selection in ATP-utilizing ACKs

Thaker *et al*.^12^, in analysis of the MtACK and EhACK structures, predicted that P_i_/PP_i_ binding does not involve the adenosine pocket and PP_i_ likely binds in a position corresponding to the position of the β- and γ-phosphates of ATP in MtACK. Our inspection of structures for the four *Mycobacterium* ACKs and the *S*. *enterica* ACKs in addition to those for MtACK and *C. neoformans* ACK showed that the opening to the adenosine pocket is not occluded in ATP-utilizing ACKs, only in the EhACK structure. Thus, we investigated whether phosphoryl donor selection by ACK is based primarily on accessibility of the adenosine pocket.

Alterations were made to MtACK to determine if the substrate specificity could be changed from ATP to PP_i_ if the adenosine pocket was occluded. Catalysis was greatly reduced (~50–150 fold) in the enzyme variants and this was accompanied by increases in the *K*_*m*_ values for both acetate and ATP in the acetyl phosphate-forming direction of the reaction. Gorrell *et al*.^16^, using tryptophan fluorescence quenching, found that domain closure occurs upon nucleotide binding. Thus, the effects of these alterations on MtACK activity may be complicated to interpret as reduced catalysis could be due to inefficient utilization of ATP and to an influence in domain closure. Notably though, substrate specificity did not change and the MtACK variant was unable to utilize PP_i_ as a substrate. Thus, conversion of ATP-dependent MtACK to a P_i_/PP_i_-dependent enzyme could not be achieved by simple closure of the adenosine pocket.

MtACK has a broad NTP substrate range^15, 17^ with a preference for ATP and is highly active in both the acetate- and acetyl phosphate-forming directions. Ingram-Smith *et al*.^15^ examined the roles of conserved active site residues in NTP substrate selection in MtACK, and found that Gly^331^ in the ADENOSINE motif exerted a strong influence. Asn^211^ in the PHOSPHATE2 motif and Gly^239^ in the LOOP3 motif were found to be important for enzymatic activity but did not play a substantial role in NTP preference.

Yoshioka *et al*.^10^ studied four residues in the ADENOSINE motif and one residue in the PHOSPHATE2 motif of *E. coli* ACK for their role in ATP versus PP_i_ substrate determination. The candidate residues Asn^213^, Gly^332^, Gly^333^, Ile^334^ and Asn^337^ were altered to the respective residues present in EhACK (Thr, Asp, Gln, Met, and Glu, respectively) and the ability of the enzyme variants to utilize PP_i_ in place of ATP was examined. All five variants displayed increased *K*_*m*_ for ATP and decreased catalysis but none was able to utilize PP_i_.

Yoshioka *et al*.^10^ also examined the distribution of the *E. coli* ACK candidate residues and the corresponding residues in EhACK among 2625 ACK homologs. They suggested that Asn^337^ (Glu^327^ of EhACK) is most important in determining substrate selection as it is present in the ten ACK sequences most closely related to EhACK. However, their kinetic results with the Asn^337^ variants are inconclusive in this regard, although the kinetic results for this and other variants do strongly support a major role in ATP binding for the ADENOSINE motif but do not delineate specific residues responsible for determining ATP versus PP_i_ utilization.

### Substrate selection in PP_i_-dependent EhACK

As a converse to our experiments with MtACK, we altered residues blocking the opening to the adenosine pocket in EhACK to reduce the occlusion and evaluated the enzyme’s ability to utilize P_i_/PP_i_ versus ATP/ADP. The EhACK variants exhibited decreased activity with P_i_ and PP_i_, much as we expected. Although catalysis was reduced for the Q^323^G-M^324^I and Q^323^A-M^324^A variants, further opening of the entrance to the adenosine pocket in the D^272^A-R^274^A-Q^323^G-M^324^I variant almost completely eliminated activity. This suggested that as the opening to the adenosine pocket increases, P_i_ and PP_i_ may still bind but their positioning may be suboptimal.

Although the enzyme variants were still unable to utilize ATP as a substrate, ATP and ADP did inhibit enzyme activity. The level of inhibition increased as the opening to the adenosine pocket was expanded, especially for the D^272^A-R^274^A-Q^323^G-M^324^I variant. This suggested that ATP and ADP could now enter the adenosine pocket and interfere with P_i_ binding. Such an interpretation of these results is supported by the observation that ATP inhibition is competitive versus P_i_.

**Figure 6:**
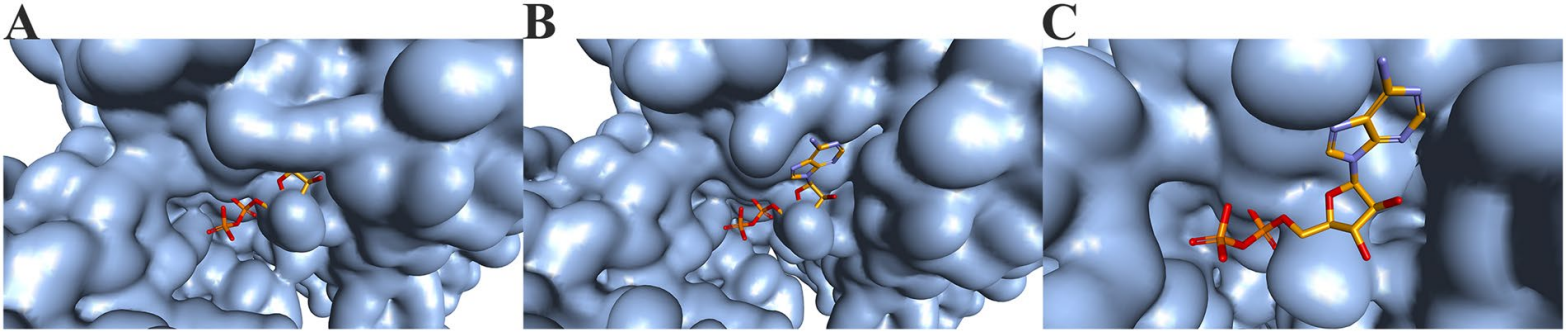
*In silico* modeling of the adenosine binding pocket of EhACK variants. Models were built using Accelrys Discovery Studio version 3.5 (Biovia). ADP binding in MtACK was superimposed into the EhACK structure models. (**A**) Q^323^G-M^324^I variant. (**B**) D^272^A-R^274^A-Q^323^G-M^324^I variant. (**C**) ΔG^203^- D^272^A-R^274^A-Q^323^G-M^324^I variant.

The similar behavior of the Q^323^G-M^324^I and Q^323^A-M^324^A variants with respect to inhibition by ATP indicated that the increased binding (as judged by IC_50_ values) must be due to expanding the entrance to the adenosine pocket rather than a specific interaction between the altered residues and ATP. Models of the enzyme variants indicated that these alterations to the adenosine pocket would result in decreased impairment of ATP binding (Fig. 6). In particular, deletion of G^203^ to shorten the PHOSPHATE2 loop combined with alteration of the ADENOSINE motif and removal of the D^272^-R^274^ salt bridge should allow the pocket to accommodate ATP well (Fig. 6C). Although the alterations made to EhACK appeared to increase the enzyme’s ability to bind ATP, as indicated by the inhibition results, ATP was still not an effective substrate.

### Other possible PP_i_-dependent ACKs

Using a BLASTp search of the non-redundant protein sequence database at NCBI with EhACK as the query sequence, we identified a small number of putative ACK sequences that may also be PP_i_-dependent or require a substrate other than ATP or PP_i_. Several of these putative ACK sequences came from metagenome analyses of anaerobic digestors. However, there were four putative ACK deduced amino acid sequences that came from draft genomes for the bacteria *Ornatilinea apprima*, *Longilinea arvoryzae*, *Flexilinea flocculi*, and *Leptolinea tardivitalis* ^18–21^. These bacteria are all obligate anaerobes from the phylum *Chloroflexi* within the family *Anaerolineaceae*. Three of the four produce acetate as a main product from glucose fermentation; *L. arvoryzae* also produces acetate as a primary fermentation product but from growth on sucrose instead of glucose. Little else is known about these bacteria beyond their initial characterization for recognition as new species.

**Figure 7:**
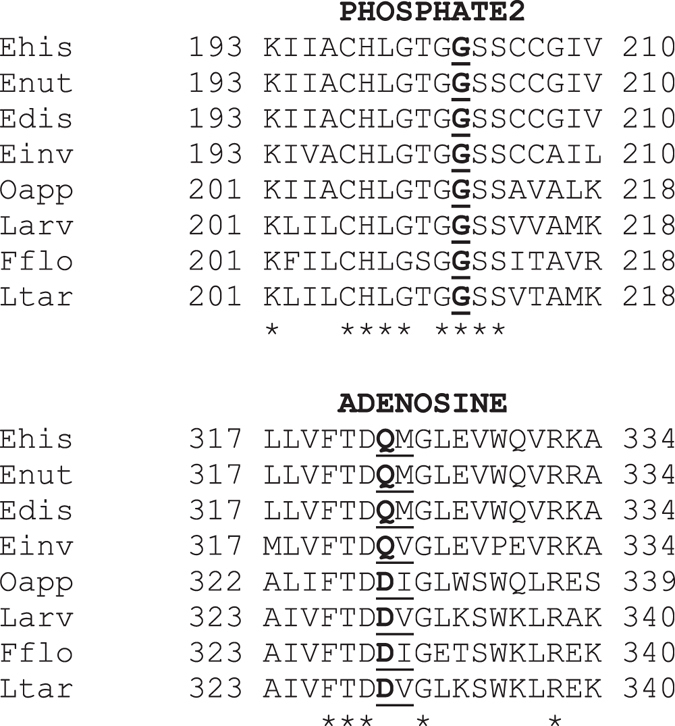
Partial alignment of putative PP_i_-ACK amino acid sequences. Sequences were aligned using Clustal Omega. The PHOSPHATE2 and ADENOSINE motifs are shown. The full alignment is provided in the Supplemental Information (Supplemental Figure S3). Abbreviations and sequence accession numbers: Ehist, *E. histolytica*, XP_655990.1; Enut, *Entamoeba nuttalli*, XP_008860710.1; Edis, *Entamoeba dispar*, XP_001741606.1; Einv, *Entamoeba invadens*, XP_004254504.1; Oapp, *Ornatilinea apprima*, WP_075061087.1; Larv, *Longilinea arvoryzae*, WP_075074878.1; Fflo, *Flexilinea flocculi*, WP_062279690.1; Ltar, *Leptolinea tardivitalis*; WP_062422928.1.

Alignment of these putative ACK sequences with those of the four *Entamoeba* ACK sequences (those from *E. histolytica*, *Entamoeba nuttalli*, *Entamoeba dispar*, and *Entamoeba invadens*) revealed two key findings. Within the PHOSPHATE2 motif, all eight of these sequences have the extended loop containing the second Gly residue (Fig. 7). These are the only putative ACK sequences identified to have this extended PHOSPHATE2 loop (see Supplemental Figure S1 for comparison). Within the ADENOSINE motif, all of these sequences have a conserved Asp residue (immediately adjacent to Q^323^-M^324^ of EhACK) that is not conserved in any other ACK sequences (all of which have Ala or Gly at the equivalent position, as shown in Fig. 1). Interestingly, these bacterial ACKs have a conserved Asp at the equivalent position to Gln^323^ of PP_i_-dependent EhACK and the other *Entamoeba* ACKs. A completely conserved Gly resides at this position (Gly^331^ of MtACK) in all ATP-dependent ACKs. Whether this indicates that these enzymes are neither ATP-dependent nor PP_i_-dependent, or whether there is some flexibility in the identity of the residue at this position in PP_i_-dependent ACKs is unknown.

## Conclusions

Our results demonstrate that phosphoryl donor specificity in ACK is mediated not just by access to the adenosine binding pocket but by other elements as well, as simple opening or occlusion of the entrance to this pocket was not sufficient to alter substrate specificity. This suggests that the active sites of the ADP/ATP-dependent and P_i_/PP_i_-dependent enzymes have evolved to optimize utilization of their preferred substrate at the expense of the ability to use alternative substrates, and thus better suit their biological function.

## Materials and Methods

### Materials

Chemicals were purchased from Sigma-Aldrich, VWR International, Gold Biotechnology, Fisher Scientific, and Life Technologies. Oligonucleotide primers were purchased from Integrated DNA Technologies.

### Site-directed mutagenesis

Site-directed mutagenesis of the *E. histolytica ack (ehack)* and *M. thermophila ack (mtack)* genes was performed according to manufacturer’s instructions with the QuikChange II kit (Agilent Technologies, CA, USA). The altered sequences were confirmed by sequencing at the Clemson University Genomics Institute. Mutagenesis primers used are shown in Supplemental Table S2.

### Recombinant protein production

EhACK and its variants were produced in *Escherichia coli* strain YBS121 *∆ack ∆pta* carrying the pREP4 plasmid containing the *lacI* gene and purified as described in Fowler *et al*.^9^. MtACK and its variants were produced in *E. coli* Rosetta2 (DE3) pLysS and purified as described in Fowler *et al*.^9^. Purified enzymes were dialyzed overnight in 25 mM Tris-HCl, 150 mM NaCl, and 10% glycerol (pH 7.4), aliquoted, and stored at −80 °C. Recombinant enzymes were examined by SDS-PAGE and estimated to be greater than 95% pure. Protein concentration was measured by absorbance at 280 nm using Take3 micro-volume plate (Biotek, VT, USA).

### Determination of kinetic parameters

Kinetic parameters for the EhACK enzymes were determined using the colorimetric hydroxamate assay for the acetyl phosphate-forming direction^2, 3, 17^ and the reverse modified hydroxamate assay^9, 22^ for the acetate-forming direction as previously described^9^. In the acetyl phosphate-forming direction, activities for EhACK and its variants were assayed in a mixture containing 100 mM morpholinoethanesulfonic acid (pH 5.5), 5 mM MgCl_2_, and 600 mM hydroxylamine hydrochloride (pH 7.5) with varying concentrations of acetate and sodium pyrophosphate. Reactions were performed at 45 °C. For the acetate-forming direction, kinetic parameters were determined in a mixture of 100 mM Tris-HCl (pH 7.0) and 10 mM MgCl_2_ with varying concentrations of sodium phosphate and acetyl phosphate. Enzymatic reactions were performed at 37 °C.

The acetyl phosphate produced (acetyl phosphate-forming direction) or remaining (acetate-forming direction) was reacted with hydroxylamine to produced acetyl hydroxamate, which was then was converted to a ferric hydroxamate complex by reaction with an acidic ferric chloride solution, making the solution change from a yellow to brownish red color that can detected spectrophotometrically at 540 nm. Acetyl phosphate formation or depletion was determined by measuring the absorbance at 540 nm with an Epoch microplate spectrophotometer (Biotek) and comparison to an acetyl phosphate standard curve. Kinetic data were fit to the Michaelis-Menten equation by nonlinear regression using KaleidaGraph (Synergy Software) for determination of apparent kinetic parameters.

Similarly, kinetic parameters for MtACK and its variants were determined using the hydroxamate assay^2, 3, 17^ and the reverse modified hydroxamate assay^9, 22^ as previously described. In the acetate-forming direction, enzyme activities were determined in 100 mM Tris (pH 7.5) with varying concentrations of MgADP and acetyl phosphate. For the acetyl phosphate-forming direction, kinetic parameters were determined in 100 mM Tris (pH 7.5) and 600 mM hydroxylamine (pH 7.5) with varying concentrations of acetate and MgATP. Enzymatic reactions were performed at 37 °C.

### Determination of inhibition parameters

Inhibition of EhACK by ATP and ADP and of MtACK by PP_i_ was determined using the hydroxamate and reverse modified hydroxamate assays as described above. All inhibition assays were performed using substrates at their *K*_*m*_ concentrations, with the exception of acetyl phosphate which was used at a concentration of 2 mM for all reactions in the acetate-forming direction. The half maximal inhibitory concentrations (IC_50_ values) were determined using PRISM 5 (Graphpad Software). ATP’s mode of inhibition of wild-type EhACK was determined by measuring enzymatic activity in the acetate forming direction in a four by seven matrix of varied ATP and P_i_ concentrations, with other substrate concentrations kept constant.

### ACK sequence alignment, ConSurf analysis, and structural modeling

ACK sequences were obtained from NCBI. Accession numbers are as follows: *E. histolytica*, PDB ID 4H0O; *Methanosarcina thermophila*, PDB ID 1TUY; *Cryptococcus neoformans*, PDB ID 4H0P; *Salmonella typhimurium*, PDB ID 3SLC; *Thermotoga maritima*, PDB ID 2IIR; *Mycobacterium smegmatis*, PDB ID 4IJN; *Mycobacterium avium*, PDB ID 3P4I; *Mycobacterium paratuberculosis*, PDB ID 3R9P; *Mycobacterium marinum* PDB ID 4DQ8. Sequences alignments were performed using Clustal Omega^23–25^. ACK structures were downloaded from Protein Data Bank (PDB): 4H0O (*Entamoeba histolytica)*, 1TUY (*M. thermophila)*, 4H0P (*C. neoformans)*, and 3SLC (*S. typhimurium)*. Structure superposition and modeling were performed using Accelrys Discovery Studio 3.5 (Biovia). ConSurf analysis^26–28^ (http://consurf.tau.ac.il) was used to examine evolutionary conservation of ACK sequence and identify amino acids likely to play important structural and functional roles.

## Electronic supplementary material

Supplementary Information
