# Supplementary material for: Investigation of pyrophosphate versus ATP substrate selection in the Entamoeba histolytica acetate kinase
Source: Sci Rep. 2017 Jul 19;7:5912. doi: 10.1038/s41598-017-06156-5 (PMC5517563; doi:10.1038/s41598-017-06156-5)
Supplement: Supplementary file 1 — Supplementary Information [file 41598_2017_6156_MOESM1_ESM.pdf]

Investigation of pyrophosphate versus ATP substrate selection in the *Entamoeba histolytica* acetate kinase

Thanh Dang and Cheryl Ingram-Smith*

Supplemental Information

Supplemental Table S1. Percent identity and similarity between ACKs[a].

	Ehist	Mtherm	Cneo	Styph	Tmari	Msmeg	Mavium	Mpara	Mmari
Ehist		35/53	29/44	34/50	38/57	34/50	31/46	32/46	30/47
Mtherm	35/53		31/49	44/62	57/76	41/59	41/59	41/58	41/58
Cneo	29/44	31/49		33/49	32/48	31/47	33/47	33/47	33/48
Styph	34/50	44/62	33/49		46/67	44/58	40/55	41/55	41/55
Tmari	38/57	57/76	32/48	46/67		47/61	45/60	45/59	43/59
Msmeg	34/50	41/59	31/47	44/58	47/61		67/77	67/77	67/78
Mavium	31/46	41/59	33/47	40/55	45/60	67/77		98/98	73/84
Mpara	32/46	41/58	33/47	41/55	45/59	67/77	98/98		73/84
Mmari	30/47	41/58	33/48	41/55	43/59	67/78	73/84	73/84	

[a] Ehist, *Entamoeba histolytica*; Mtherm, *Methanosarcina thermophila*; Cneo, *Cryptococcus neoformans*; Styph, *Salmonella typhimurium*; Tmari, *Thermotoga maritima*; Msmeg, *Mycobacterium smegmatis*; Mavium, *Mycobacterium avium*; Mpara, *Mycobacterium paratuberculosis*; Mmari, *Mycobacterium marinum*.

Supplemental Table S2. Primers used for mutagenesis.

**EhACK primers**	
$Q^{323}G-M^{324}I$ F	5’GATCTTTTAGTATTTACTGATGGCATCGGATTAGAAGTTTGGCAAGTACG
$Q^{323}G-M^{324}I$ R	5’CGTACTTGCCAAACTTCTAATCCGATGCCATCAGTAAATACTAAAAGATC
$Q^{323}A-M^{324}A$ F	5’GATCTTTTAGTATTTACTGATGCCGCGGGATTAGAAGTTTGGCAAGTACG
$Q^{323}A-M^{324}A$ R	5’CGTACTTGCCAAACTTCTAATCCCGCGGCATCAGTAAATACTAAAAGATC
$D^{272}A-R^{274}A$ F	5’GGTGTTAGTGAATTATCTAGTGCTATGGCAGATATTTTACATGAAATAG
$D^{272}A-R^{274}A$ R	5'CTATTTCATGTAAAATATCTGCCATAGCACTAGATAATTCACTAACACC
G^{203} Deletion F	5’GCTTGTCATCTTGGAACAGGTTCTAGTTGTTGTGGCATTGTTAATGG
G^{203} Deletion R	5’CCATTAACAATGCCACAACAACTAGAACCTGTTCCAAGATGACAAGC
**MtACK primers**	
G331Q-I332M F	5’ GCAGTGGTCTTTACTGCACAGATGGGAGAAAACAGCGCAAGC
G331Q-I332M R	5’ GCTTGCGCTGTTTTCTCCCATCTGTGCAGTAAAGACCACTGC

Supplemental Figure S1. SDS-PAGE analysis of purified enzymes.

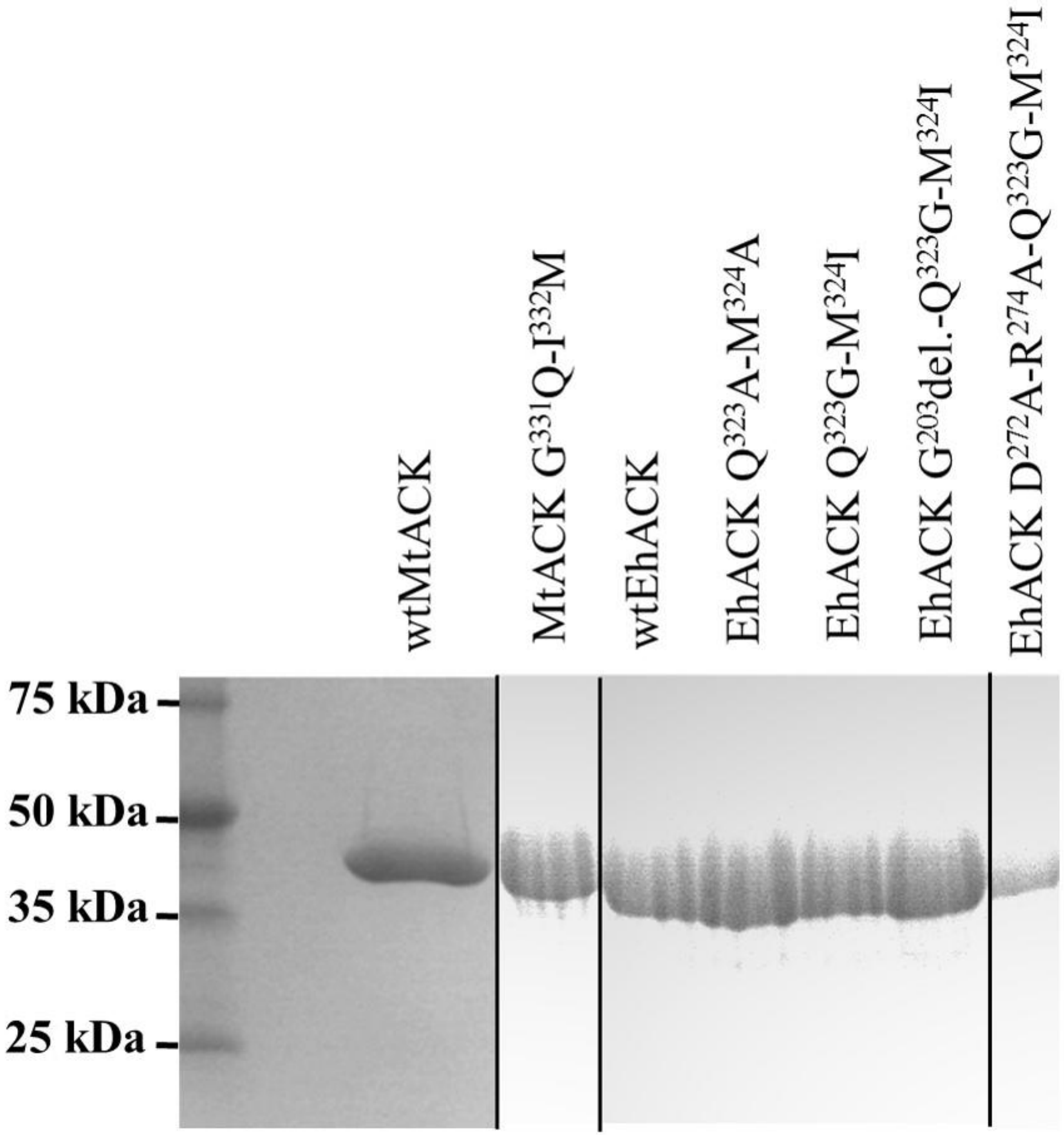

Supplemental Figure S2. ACK sequence alignment. The full ACK sequence alignment from which Figure 1 was derived is shown. Abbreviations and PDB accession numbers: Tmari, *Thermotoga maritima*, PDB ID 2IIR; Mtherm, *Methanosarcina thermophila*, PDB ID 1TUY; Mavium, *Mycobacterium avium*, PDB ID 3P4I; Mpara, *Mycobacterium paratuberculosis*, PDB ID 3R9P; Msmeg, *Mycobacterium smegmatis*, PDB ID 4IJN; Styph, *Salmonella typhimurium*, PDB ID 3SLC; Ehist, *E. histolytica*, PDB ID 4H0O; Cneo, *Cryptococcus neoformans*, PDB ID 4H0P.

```
                         ____PHOSPHATE1_____
Tmari           -------MRVLVINSGSSSIKYQLIEMEGEKVLC-------KGIAERIGIEGSRLVHRVG 46
Mtherm          -------MKVLVINAGSSSLKYQLIDMTNESALA-------VGLCERIGIDNSIITQKKF 46
Mavium          MDGSDGARRVLVINSGSSSLKFQLVDPESGVAAS-------TGIVERIGEESS------- 46
Mpara           MDGSDGARRVLVINSGSSSLKFQLVDPEFGVAAS-------TGIVERIGEESS------- 46
Msmeg           ------MVTVLVVNSGSSSLKYAVVRPASGEFLA-------DGIIEEIG--SG------- 38
Styph           ----MSSKLVLVLNCGSSSLKFAIIDAVNGDEYL-------SGLAECFHLPEARIKWKMD 49
Ehist           ------MSNVLIFNVGSSSLTYKVFCSDNIVCSG-------KSNRVNVTGTEKPFIEHHL 47
Cneo            --MPDKAEYLLAINCGSSSIKGKLFAIPSFELLANLAVTNISSSDERVKIKTTWEEGKGK 58
                         :* .* ****:.  :.                 .    .

Tmari           DEKHVIER-ELPDHEEALKLILNTLVDEKLGVIKDLKEIDAVGHRVVHGGERFKESVLVD 105
Mtherm          DGKKLEKLTDLPTHKDALEEVVKALTDDEFGVIKDMGEINAVGHRVVHGGEKFTTSALYD 106
Mavium          ---------PVPDHDAALRRAFDMLAGD--GVDLNTAGLVAVGHRVVHGGNTFYRPTVLD 95
Mpara           ---------PVPDHDAALRRAFDMLAGD--GVDLNTAGLVAVGHRVVHGGNTFYRPTVLD 95
Msmeg           ---------AVPDHDAALRAAFDELAAA--GLHLEDLDLKAVGHRMVHGGKTFYKPSVVD 87
Styph           GSKQEAALGAGAAHSEALNFIVNTILAQ---KPELSAQLTAIGHRIVHGGEKYTSSVVID 106
Ehist           NGQIIKIETPILNHPQAAKLIIQFLKEN-------HISIAFVGHRFVHGGSYFKKSAVID 100
Cneo            DSEEEADYGDKIRYASLVPILLDHLTNS---THVKKEEIKYVCHRVVHGGMHDKGIRVVK 115
                             :       .. :             :  : **.****       : .

Tmari           --EEVLKAIEEVSPLAPLHNPANLMGIKAAMKLLPGVP--NVAVFDTAFHQTIPQKAYLY 161
Mtherm          --EGVEKAIKDCFELAPLHNPPNMMGISACAEIMPGTP--MVIVFDTAFHQTMPPYAYMY 162
Mavium          --DAVIARLHELSELAPLHNPPALLGIEVARRLLPGIA--HVAVFDTGFFHDLPPAAATY 151
Mpara           --DAVIARLHELSELAPLHNPPALQGIEVARRLLPDIA--HVAVFDTGFFHDLPPAAATY 151
Msmeg           --DELIAKARELSPLAPLHNPPAIKGIEVARKLLPDLP--HIAVFDTAFFHDLPAPASTY 143
Styph           --ESVIQGIKDSASFAPLHNPAHLIGIAEALKSFPQLKDKNVAVFDTAFHQTMPEESYLY 164
Ehist           --EVVLKELKECLPLAPIHNPSSFGVIEISMKELPTTR--QYVAIDTAFHSTISQAERTY 156
Cneo            GHEEGLMEMDKLSEFAPLHNHRAVLAVKSCIDALPHHT--SLLLFDTIFHRTIAPEVYTY 173
                  :       .   :**:**   .  :  .   :*         :** *.  :.     *

                                                         ____PHOSPHATE2____
Tmari           AIP-YEYYEKYKIRRYGFHGTSHRYVSKRAAEILGKKLEELKIITCHIGNG-ASVAAVKY 219
Mtherm          ALP-YDLYEKHGVRKYGFHGTSHKYVAERAALMLGKPAEETKIITCHLGNG-SSITAVEG 220
Mavium          AID-RELADRWQIRRYGFHGTSHRYVSEQAAAFLDRPLRGLKQIVLHLGNG-CSASAIAG 209
Mpara           AID-RELADRWQIRRYGFHGTSHRYVSEQAAAFLDRPLRGLKQIVLHLGNG-CSASAIAG 209
Msmeg           AID-RELAETWHIKRYGFHGTSHEYVSQQAAIFLDRPLESLNQIVLHLGNG-ASASAVAG 201
Styph           ALP-YSLYKEHGVRRYGAHGTSHFYVTQEAAKMLNKPVEELNIITCHLGNG-GSVSAIRN 222
Ehist           AIP-QPYQSQY--LKFGFHGLSYEYVINSLKNVID--VSHSKIIACHLGTGGSSCCGIVN 211
Cneo            ALPPPDTELTMPLRKYGFHGLSYASIVQSLAEHLKKPSDQINVVVAHLGSG-SSSCCIKN 232
                *:            ::* ** *:  : :     :       : :. *:*.*  *   :
```

```
                        ___________LOOP3_____________________
Tmari           GKCVDTSMGFTPLEGLVMGTRSGDLDPAIPFFIMEKEG-------ISPQEMYDILNKKSG 272
Mtherm          GKSVETSMGFTPLEGLAMGTRCGSIDPAIVPFLMEKEG-------LTTREIDTLMNKKSG 273
Mavium          TRPLDTSMGLTPLEGLVMGTRSGDIDPSVVSYLCHTAG-------MGVDDVESMLNHRSG 262
Mpara           TRPLDTSMGLTPLEGLVMGTRSGDIDPSIVSYLCHTAG-------MGVDDVESMLNHRSG 262
Msmeg           GKAVDTSMGLTPMEGLVMGTRSGDIDPGVIMYLWRTAG-------MSVDDIESMLNRRSG 254
Styph           GKCVDTSMGLTPLEGLVMGTRSGDIDPAIIFHLHDTLG-------MSVDQINKMLTKESG 275
Ehist           GKSFDTSMGNSTLAGLVMSTRCGDIDPTIPIDMIQQVG-------IEK--VVDILNKKSG 262
Cneo            GKSIDTSMGLTPLEGLLGGTRSGTIDPTAIFHHTEDAASDANVGDFTVSKAEIILNKNSG 292
                 : .:**** :.: **  .**.* :**         .       :        ::.:.**

                         ___LOOP4_____
Tmari           VYGLSKGFSSDMRDIEEAALKG------DEWCKLVLEIYDYRIAKYIGAYAAAMN----- 321
Mtherm          VLGVS-GLSNDFRDLDEAASKG------NRKAELALEIFAYKVKKFIGEYSAVLN----- 321
Mavium          VVGLS--GVRDFRRLRELIESG------DGAAQLAYSVFTHRLRKYIGAYLAVLG----- 309
Mpara           VVGLS--GVRDFRRLRELIESG------DGAAQLAYSVFTHRLRKYIGAYLAVLG----- 309
Msmeg           VLGLG--GASDFRKLRELIESG------DEHAKLAYDVYIHRLRKYIGAYMAVLG----- 301
Styph           LLGLT-EVTSDCRYVEDNYAT-------KEDAKRAMDVYCHRLAKYIGSYTALMDG---- 323
Ehist           LLGVS-ELSSDMRDILHEIETRGP---KAKTCQLAFDVYIKQLAKTIGGLMVEIG----- 313
Cneo            FKALA--GTTNFGHIIQNLDPSKCSEEDHEKAKLTYAVFLDRLLNFVAQYLFKLLSEVPI 350
                . .:      :   : .             .: .   ::  :: : :.    :

                       ____ADENOSINE_______
Tmari           -GVDAIVFTAGVGENSPITREDVCSYLEFLGVKLDKQKNEETIRGKEGIISTPDSRVKVL 380
Mtherm          -GADAVVFTAGIGENSASIRKRILTGLDGIGIKIDDEKNK--IRGQEIDISTPDAKVRVF 378
Mavium          -HTDVISFTAGIGENDAAVRRDAVSGMEELGIVLDERRNLPGAKGAR-QISADDSPITVL 367
Mpara           -HTDVISFTAGIGENDAAVRRDAVSGMEELGIVLDERRNLAGGKGAR-QISADDSPITVL 367
Msmeg           -RTDVISFTAGVGENVPPVRRDALAGLGGLGIEIDDALNSAKSDEPR-LISTPDSRVTVL 359
Styph           -RLDAVVFTGGIGENAAMVRELSLGKLGVLGFEVDHERNLAARFGKSGFINKEGTRP-AV 381
Ehist           -GLDLLVFTDQMGLEVWQVRKAICDKMKFLGIELDDSLNEKSMGKKIEFLTMPSSKVQVC 372
Cneo            ESIDGLVFSGGIGEKGAELRRDVLKKLAWLGAEVDEEANNSNSGGAVKCITKEGSKLKGW 410
                   * : *:  :* :    *.     :  :* :*.  *          :.  .:

Tmari           VVPTNEELMIARDTKEIVEKIGR------- 403
Mtherm          VIPTNEELAIARETKEIVETEVKLRSSIPV 408
Mavium          VVPTNEELAIARDCVRVLGG---------- 387
Mpara           VVPTNEELAIARDCVRVLGG---------- 387
Msmeg           VVPTNEELAIARACVGVV------------ 377
Styph           VIPTNEELVIAQDASRLTA----------- 400
Ehist           VAPNDEELVILQKGKELFQF---------- 392
Cneo            VVETDEEGWMARMAKEEFGF---------- 430
                *  .:**  : :
```

Supplemental Figure S3. Alignment of putative PPi-ACK sequences. The full ACK sequence alignment from which Figure 7 was derived is shown. Abbreviations: Ehist, *E. histolytica*, XP_655990.1; Enut, *Entamoeba nuttalli*, XP_008860710.1; Edis, *Entamoeba dispar*, XP_001741606.1; Einv, *Entamoeba invadens*, XP_004254504.1; Oapp, *Ornatilinea apprima*, WP_075061087.1; Larv, *Longilinea arvoryzae*, WP_075074878.1; Fflo, *Flexilinea flocculi*, WP_062279690.1; Ltar, *Leptolinea tardivitalis*; WP_062422928.1.

```
                PHOSPHATE1
Ehis      MSNVLIFNVGSSSLTYKVFCSDN-----IVCSGKSNRVNVTGTEKPFIEHHLNGQIIKIE 55
Enut      MSNVLIFNVGSSSLTYKVFCSDN-----IVCNGKANRVNVTGTEKPFIEHHLNGKIIKIE 55
Edis      MSNVLIFNVGSSSLTYKVFCSDK-----IVCSGKANRVNVTGTTKPFIEHHLNGKVIKVE 55
Einv      MPHILVFNVGSSSLTYKLFENTK-----EIIKGKANRVNVTGAELPFIEHHINGKTITIE 55
Oapp      -MNLLVFNCGSSSLNYKVFSGDSNSTAQITAKGKAHRVGVKGSDPSFIEHHLSNQTVKET 59
Larv      -MNILIFNCGSSSQGFKVYQTSGHDQPVVLVSGKAKNVATQTRADAFIEWKSKISAGSQK 59
Fflo      -MNILVFNCGSSSQGFKVYEVDQNHAEKVVISGKAKNVAARTQSQPYLFWNMNDKTEQKF 59
Ltar      -MNILIFNCGSSSQGFKLYQKEYGTTPILVAAGKARNVATKTRADSCLDWTAGSQKGSVN 59
            ::*:** ****  :*::             **:..* .     . :      .

Ehis      TPILNHPQAAKLIIQFLKENHISIAFVGHRFVHGGSYFKKSAVIDEVVLKELKECLPLAP 115
Enut      TPILNHPQAAKLIIQFLKENHISIAFVGHRFVHGGSYFKKSAIIDEVVLKELKECLPLAP 115
Edis      TPTLNHQQAAEFIIQFLKENHVSIAFVGHRFVHGGSYFKRSAIIDEAVLKELKECLPLAP 115
Einv      TGPLNHQEAARLIIKFLKENKFTIDIVGHRFVHGGSYFKTSAVIEGPVLKELKSCIPLAP 115
Oapp      QPLETHAQAAERVLQNLRDHQIPIDAVGHRFVHGGAYFKESALLTEDTLARLTECLPLAP 119
Larv      TDLSSHRQAAGKIIAILKELQVSVDAIGHRFVHGGTFFDKTVQIDPPVLQKLQQCLPFAP 119
Fflo      CDLSSHRLAAQEIIGILNAKGIQPDAIGHRFVHGGKLFQQTTRIDTNTRNLLIQCLPLAP 119
Ltar      VELPSHREAARQILALLRKSNLSVDAIGHRFVHGGDVFQHTTRIDKAVLAGLKGCFPLAP 119
              .*  **  ::  *.   .    :********  *. :. :   .   *  *:*:**

Ehis      IHNPSSFGVIEISMKELPTTRQYVAIDTAFHSTISQAERTYAIPQPYQS--QYLKFGFHG 173
Enut      IHNPSSFGVIEMSMKELPTTRQYVAIDTAFHSTISQAERTYAIPQPYQS--QYLKFGFHG 173
Edis      IHNPSSFSVIEVSMKELPTTKQYVAIDTAFHSTISQAERTYAIPQPYQS--QYLKFGFHG 173
Einv      IHNPASYSVIEVALTELPNTKQYVAIDTAFHSTINKTQRTYAIPEPFQS--QYLKFGFHG 173
Oapp      IHNPNSMSVIYTCLEHQPGCPQYVTFDTAFHAALPPEAYTYAVPQSIRDTHTYRRFGFHG 179
Larv      IHNPNSYSVIEVCLEQFPDVPQFAVFDTAFHARMPEVSKQYAIPRDLVEKYGYYKYGFHG 179
Fflo      IHNPNSFSVIEVCEQLLPSIPQYAVFDTAFHSQMPESSARYAIPGSIAEKFGFRKYGFHG 179
Ltar      IHNPNSYSVIEVCRELLPDAAQFAVFDTAFHANMPAESRQYALPRELVQENGYRKYGFHG 179
          **** * .**  .    *   *:..:*****: :      **:*    .   : ::****

                         PHOSPHATE2                  LOOP3
Ehis      LSYEYVINSLKNVID--VSHSKIIACHLGTGGSSCCGIVNGKSFDTSMGNSTLAGLVMST 231
Enut      LSYEYVINSLKNVID--VSHSKIIACHLGTGGSSCCGIVNGKSFDTSMGNSTLAGLVMST 231
Edis      LSYEYVINSLKNVID--VSHSKIIACHLGTGGSSCCGIVNGKSFDTSMGNSTLAGLVMST 231
Einv      LSYEFVLTSLKERMD--VDKLKIVACHLGTGGSSCCAILNGKSYDTSMGNSTLAGLVMST 231
Oapp      LSYHFVTQAAGPFLETPFSESKIIACHLGTGGSSAVALKNGVPLDTSMGFTPLPGLIMST 239
Larv      LSYQYVSSRMLELMGKPLEELKLILCHLGTGGSSVVAMKNGLPLDTSMGYSPLAGLVMST 239
Fflo      LSYQYVSTKTAQLIGKPLQNSKFILCHLGSGGSSITAVRDGKSIDTSMGYSPLAGLVMSS 239
Ltar      LSYQYVSARTAEYLGRPLEELKLILCHLGTGGSSVTAMKDGRSMDSSMGYSPLPGLVMST 239
          ***.:*       :   ... *:: ****:****  .: :* . *:*** :.*.**:**:
```

```
                                                        LOOP4
Ehis      RCGDIDPTIPIDMIQQVGIEKVVD-ILNKKSGLLGVSELSSDMRDILHEIETRGPKAKTC 290
Enut      RCGDIDPTIPIDMIQQVGIEKVVD-ILNKKSGLLGVSELSSDMRDILHEIEIRGPKAKTC 290
Edis      RCGDIDPTIPIDMIQQVGVERVVD-ILNKRSGLLGVSELSSDMRDILHEIEIKGPKAKTC 290
Einv      RCGDIDPSIPINIVEQIGIQKTVD-LLNKRSGLFGVSETSCDIRDLLKEIKENGQKAEKC 290
Oapp      RTGDLDAQIPIQLLKEGKSPKEIETLLNKKSGLLGISQFSSDLRDILARVE----QDPNA 295
Larv      RSGDIDPEIVLEMIRNGSSPDEVSQILNNRSGLIGLSGFSSSLPEIIEASE---KGNADC 296
Fflo      RSGDLDPEIILDLVRSGYSADEVSRILNRESGLIGLSGFSSNLAEIIDAAE---SGNVSC 296
Ltar      RCGDLDPEIVLEMIRRGSSVDDVEFILNNQSGLIGLSGYSSNLEEVIAEGE---KGNEDC 296
          * **:*. * ::::.       :. :**..***:*:*  *..: :::   :        .

                                           ADENOSINE
Ehis      QLAFDVYIKQLAKTIGGLMVEIGGLDLLVFTDQMGLEVWQVRKAICDKMKFLGIELDDSL 350
Enut      QLAFDVYIKQLAKTIGGLMVEIGGLDLLVFTDQMGLEVWQVRRAICDKMKFLGIELDDSI 350
Edis      QLAFDVYIKQLAKTIGGLMVEIRGLDLLVFTDQMGLEVWQVRKAICDKMKFLGIELDNSL 350
Einv      ALAFDLYINQLTKTIGGLMVEIGGLDMLVFTDQVGLEVPEVRKAVCNKLEFLGVELDSEK 350
Oapp      HLAFEMAVHRLVKYIGAYAVLLGGLDALIFTDDIGLWSWQLRESVCQGLTWCGIAIDPTA 355
Larv      QLAYDVYAHRLETYLGAYTWLLDGADAIVFTDDVGLKSWKLRAKVCGGVQNLGVEIDAAK 356
Fflo      QIAFDVYAQRLMEYMGAFYWLLNGADAIVFTDDIGETSWKLREKLFGGKDLLGVKLDQEL 356
Ltar      RLAFDVYAHRLQLYLGAFFWLLNDADAIVFTDDVGLKSWKLREKVCRGVENLGILLDADA 356
           :*:::  ::*   :*.    : . * ::***::*    ::*  :       *: :*

Ehis      NEKSMGKKIEFLTMPSSKVQVCVAPNDEELVILQKGKELFQF------ 392
Enut      NEKSMGKKIEFLTMPSSKVQVCVAPNDEELVILQKGKELFQF------ 392
Edis      NEKSMGKKIEFLTTPSSKVQVCVAPNDEELVILQKGKELFQF------ 392
Einv      NEKSRGKEIEFISTEKSRMKICVVPNDEELVILKKGSELFQFCN---- 394
Oapp      NREAPYDRITPIEAPDSRARVLVIPTDEEWVIGQEGFALLQEGRHAYH 403
Larv      NVNAPLDRASRVSSARSKTQIWVMPTDEESVILQEILAQFCLA----- 399
Fflo      NRQATGSKPSCISQEGSKTQIWVIPTDEEIVILNEVRAIIG------- 397
Ltar      NRLALPDQITCFSSPASRTRLLTVPTDEEQVILQEVLSQLEQA----- 399
          *  :  ..   .    *: :: . *.*** ** ::    :
```
